# A Case of Colon Leiomyosarcoma Arising from the Muscularis Mucosae: A Case Report and Literature Review

**DOI:** 10.70352/scrj.cr.25-0146

**Published:** 2025-07-02

**Authors:** Yuta Kasagi, Masahiko Sugiyama, Rena Yokomizo, Munehide Terashi, Taichiro Nagai, Naomichi Koga, Ayako Iwanaga, Yasue Kimura, Yutaka Koga, Kenichi Taguchi, Masaru Morita

**Affiliations:** 1 Department of Gastroenterological Surgery, NHO Kyushu Cancer Center, Fukuoka, Japan; 2 Department of Pathology, NHO Kyushu Cancer Center, Fukuoka, Japan

**Keywords:** colon leiomyosarcoma, muscularis mucosae, therapeutic strategies, literature review

## Abstract

**INTRODUCTION:**

Colon leiomyosarcoma (CLMS) is an extremely rare neoplasm and the information regarding its clinical characteristics and specific treatment was still unclear.

**CASE PRESENTATION:**

A 73-year-old female who had been diagnosed with transverse CLMS underwent laparoscopic surgery. The resected specimen showed that the tumor contained proliferation of spindle-shaped cells and arranged in fascicular pattern, which was immunohistochemically positive for smooth muscle actin and desmin. Moreover, the tumor bottom was not continuous with the muscularis propria and had a clear border in the submucosa layer. According to these findings, we finally diagnosed it as primary CLMS arising from the muscularis mucosa (MM). No recurrence was noted 24 months after surgery.

**CONCLUSIONS:**

This literature demonstrates CLMS arising from MM and suggests that its pathological diagnosis is associated with disease prognosis.

## Abbreviations

CLMS
: colon leiomyosarcoma
GI
: gastrointestinal
GIST
: gastrointestinal stromal tumor
IHC
: immunohistochemistry
LMS
: leiomyosarcoma
MM
: muscularis mucosae
MP
: muscularis propria
p
: pathological
SM
: submucosa

## INTRODUCTION

LMSs usually originate from smooth muscle, which is widespread in the body. LMSs are most common in the retroperitoneum (including the pelvis), large blood vessels (principally the inferior vena cava), and the lower extremities.^1)^ GI LMSs are uncommon neoplasms that arise from the MM or MP, and CLMS is an extremely rare neoplasm, accounting for less than 0.1% of all colorectal malignancies.^2)^

Immunohistochemically, true GI LMS expresses smooth muscle actin (SMA) and desmin without the expression of GIST markers (CD117, CD34, and DOG1) and KIT mutations, which allow LMS to be distinguished from other mesenchymal neoplasms.^3)^

Before the introduction of the oncogenic role of KIT by IHC in 1998, most of the GI LMSs were wrongfully diagnosed as leiomyoma or leiomyoblastomas, particularly GISTs.^4)^

GISTs are the most common mesenchymal GI malignancies, with an incidence of 1%–3% of all GI malignancies. Moreover, GI LMS is an extremely rare cancer representing 3%–6% of all GI mesenchymal tumors.^5)^ In the past, before the diagnosis of KIT mutations by IHC, the prognosis of CLMS was generally considered to be a benign tumor that displayed optimism with a low propensity for recurrence and distant metastasis.^6)^ Later on, the number of reports on true CLMS has rapidly decreased, and those reports have shown that frequent recurrences and distant metastasis have been observed in the CLMS.^7)^

Due to the paucity of data about CLMS, the information regarding its clinical characteristics, specific treatment, and influencing prognostics factors is still unclear. Herein, we present a case of CLMS that arose from MM, and attempt to show its clinicopathological characteristics, therapeutic maneuvers, and prognostics predictive factors by reviewing previously published manuscripts.

## CASE PRESENTATION

**Fig. 1:**
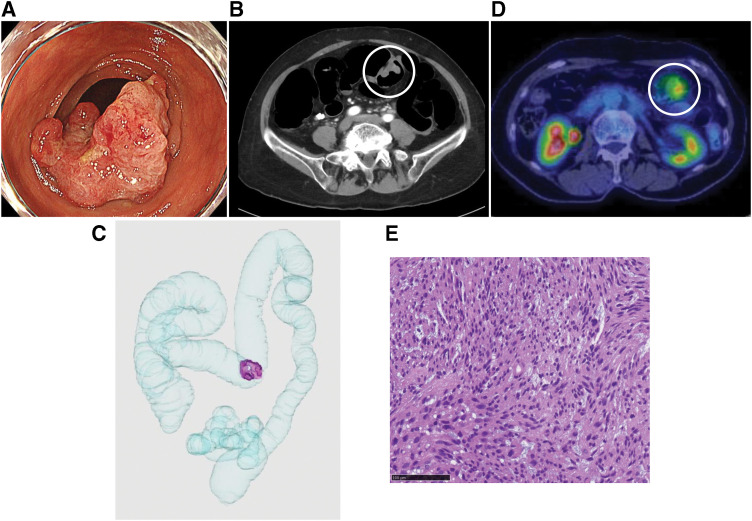
(**A**) Colonoscopy findings: A Bormann’s type 2 tumor was located in the transverse colon. (**B**, **C**) CT and CT colonography findings: CT and colonography revealed a 3-cm intradural mass in the middle transverse colon (circle and purple-colored mass). (**D**) PET-CT findings: PET-CT showed a mildly enhanced mass in the transverse colon (circle); there was no evidence of distant metastasis. (**E**) Pathological examination: The pathological finding revealed the spindle-shaped tumor cells (100 μm, scale bar). PET, positron emission tomography

**Fig. 2:**
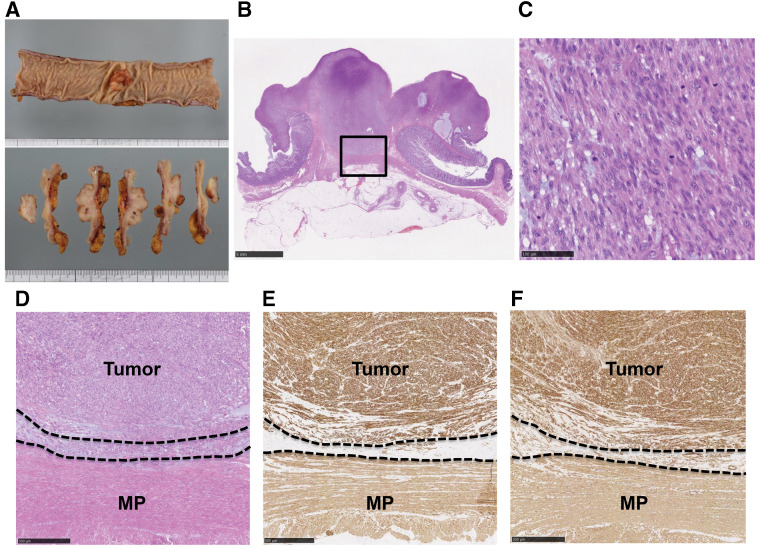
(**A**) The resected specimen: The tumor dimension was 3.0 × 2.4 cm. (**B**, **C**) H&E staining images: Macroscopic finding (**B**, 5 mm, scale bar) and microscopic finding (**C**, 100 μm, scale bar): The tumor contained a proliferation of spindle-shaped cells, arranged in a fascicular pattern. (**D**) Microscopic H&E findings (500 μm, scale bar, square in **B**): There was a border between the tumor and the MP in the submucosa layer. (**E**, **F**) Microscopic IHC findings (100 μm, scale bar, square in **B**): The tumor was immunohistochemically positive for desmin (**E**) and SMA (**F**), and the desmin staining revealed that tumor bottom was not continuous with MP (**E**). H&E, hematoxylin and eosin; IHC, immunohistochemistry; MP, muscularis propria

A 73-year-old female presented to our institution for a total screening colonoscopy. She was found to have a Bormann’s type 2 tumor located in the transverse colon (**Fig. 1A**). CT colonography showed a 3-cm intradural mass in the middle transverse colon (**Fig. 1B** and **1C**), and PET-CT revealed a mildly enhanced mass (**Fig. 1D**). There was no evidence of distant metastasis in these imaging examinations. The IHC findings showed the spindle-shaped tumor cells (**Fig. 1E**) that were positive for SMA, desmin, and negative for KIT, CD34 (data not shown). According to the IHC findings, the tumor was diagnosed as a CLMS. At that time, she sometimes suffered from abdominal pain. We were concerned that the symptoms indicated the pre-obstruction due to the tumor, and decided to perform laparoscopic surgery with lymph node (LN) dissection. The resected specimen (**Fig. 2A** and **2B**) showed that the tumor contained a proliferation of spindle-shaped cells arranged in a fascicular pattern (**Fig. 2C**), which was immunohistochemically positive for desmin (**Fig. 2E**), SMA (**Fig. 2F**), h-caldesmon, CAM5.2, and EMA (data not shown), and negative for c-kit, AE1/AE3, CD34, and S-100 (data not shown). The Ki67 and MIB1 labeling indices were both at 70% in the hot spot. Moreover, the tumor bottom was not continuous with MP and had a clear border in the SM layer (**Fig. 2D**–**2F**). According to these findings, we finally diagnosed it as primary CLMS arising from MM.

After the surgery, she did not receive adjuvant chemotherapy. We performed close follow-up and regular imaging examinations, and there was no evidence of recurrence 24 months after the resection.

## DISCUSSION

CLMSs are extremely rare neoplasms, and the prognosis has generally been considered poorer than that of other colon malignancies.^7)^ Most of them have been described as case reports, and their clinical characteristics and specific treatment are still unknown.

We searched the PubMed database using the MeSH terms “leiomyosarcoma” and “colon.” Only English-language reports published after 2000 were included. All cited references were also reviewed. The search yielded 200 publications; there were 51 published cases of CLMS that underwent surgery and were clearly diagnosed by IHC staining. **Table 1** summarizes these data, including our case.^2,4,5,8–39)^ The patients consisted of 32 males and 20 females, with a median age of 65 years (range, 24–94 years). There were 26 right-side colon cases and 26 left-side cases, most frequently in the sigmoid colon (19 cases). The median tumor size was 5.7 cm (median, 1–23 cm). There were 31 cases of localized disease and 11 cases with distant lesions (10 cases were not described).

**Table 1:** Summary of all selected cases and our case

Case	Ref. (year)	Age	Sex	Site	Diameter (cm)	Mural involvement	Metastasis	Adjuvant	Recurrence/month	Outcome	F/U period (month)
1	2000^2)^	54	F	D	3.2	NA	NA	NA	NA	D/D	37
2	2000^2)^	61	M	A	4.2	NA	NA	NA	–	Alive	141
3	2000^2)^	75	M	A	6.5	NA	NA	NA	NA	D/D	6
4	2000^2)^	76	F	C	7.8	NA	NA	NA	NA	D/D	7
5	2000^2)^	36	F	S	6.5	NA	NA	NA	Lung/32	Dead	38
6	2000^2)^	66	M	A	NA	NA	NA	NA	Liver/NA	Dead	19
7	2000^2)^	41	M	C	7.5	NA	NA	NA	Humerus/11	Alive	185
8	2004^8)^	65	M	D	10	SE	NA	NA	Visceral/NA	Dead	28
9	2004^9)^	67	F	T	5.7	NA				Alive	12
10	2007^10)^	77	F	S	NA	NA		NA	Peritoneum/4	NA	4
11	2007^10)^	52	M	S	NA	NA	Liver	NA	NA	NA	NA
12	2011^11)^	70	F	S	3.7	NA	NA	NA	Ileum/4	Dead	4
13	2011^11)^	56	M	C	NA	NA	NA	IFM + EPI	Liver/6	Alive	7
14	2012^12)^	66	F	S	3	NA	Liver		Liver/36	Dead	58
15	2013^13)^	94	F	D	25	SS		NA	Liver/4	Dead	7
16	2013^13)^	56	M	S	1	SM	LN	NA	–	Alive	60
17	2013^13)^	78	F	S	8.5	SS		NA	Lung/10	Dead	16
18	2013^13)^	87	M	T	11	MP		NA	–	D/D	2
19	2014^14)^	66	F	T	4	NA		IFM + DOX	–	Alive	33
20	2014^15)^	65	M	S	NA	NA		–	–	Alive	12
21	2015^4)^	46	M	T	11.8	SS		–	Peritoneum/9	Alive	13
22	2015^16)^	89	F	A	4.5	NA	Liver	+/NA	–	NA	NA
23	2015^17)^	54	M	A	13	SS		NA	Local/6	Alive	6
24	2015^18)^	59	M	A	10	NA		+/NA	–	Alive	8
25	2015^19)^	68	F	NA	NA	NA	Liver	DOX	+/NA	Dead	18
26	2015^19)^	24	F	S	NA	NA	Liver	–	+/NA	Dead	24
27	2015^19)^	69	M	S	NA	NA	Lung	–	Liver/NA	Dead	7
28	2015^19)^	30	M	NA	4	NA	Liver	IFM + DOX	+/NA	Dead	18
29	2016^20)^	82	M	C	2.2	SS	–	NA	–	Alive	14
30	2016^21)^	51	M	D	4	NA	–	NA	–	Alive	31
31	2017^22)^	44	M	T	8.5	MP	–	–	–	Alive	6
32	2018^23)^	55	F	A	8	SE	–	NA	–	Alive	5
33	2018^24)^	57	F	S	NA	NA	–	NA	–	Alive	NA
34	2018^24)^	88	M	A	6.5	MP	Liver	–	–	Dead	2
35	2019^5)^	46	M	S	4.2	SM	–	–	–	Alive	17
36	2020^25)^	53	M	S	3.5	MP	–	–	–	Alive	15
37	2020^26)^	71	M	T	4.5	SM	–	–	–	NA	NA
38	2020^27)^	76	F	T	5.5	MP	LN	NA	–	Alive	18
39	2020^28)^	49	F	T	5	NA	–	–	LN/14	Alive	22
40	2020^29)^	68	M	D	4	MP	–	–	–	Alive	NA
41	2021^30)^	48	M	S	7.2	NA	–	NA	–	Alive	60
42	2021^30)^	49	M	S	4	NA	Peritoneum	–	Lung/6	Alive	6
43	2021^31)^	73	M	S	5.5	NA	–	IFM+DOX	–	Alive	6
44	2021^32)^	65	F	S	16	NA	–	NA	–	Alive	1
45	2021^33)^	59	M	C	2	SM	–	+/NA	–	Alive	6
46	2021^34)^	72	M	T	23	MP	–	–	NA	NA	–
47	2022^35)^	74	M	S	8.7	NA	Lung	NA	NA	Alive	NA
48	2022^36)^	51	M	S	6	MP	–	GEM + DTX	–	Alive	12
49	2022^37)^	74	F	D	4	MP	LN, liver	NA	Lung/10	Alive	10
50	2023^38)^	38	M	D	7.5	NA	–	NA	Liver/10	Alive	52
51	2023^39)^	30's	M	T	6.5	MP	LN	–	–	Alive	2
Ours	2024	73	F	T	3	SM	–	–	–	Alive	24

A, ascending colon; Adjuvant, adjuvant chemotherapy; C, caecum; D/D, dead of difference disease; D, descending colon; DOX, doxorubicin; DTX, docetaxel; EPI, epirubicin; F/U, follow up; F, female; GEM, gemcitabine; IFM, ifosfamide; LN, lymph node; M, male; MP, muscularis propria; NA, not available; Ref., reference; S, sigmoid colon; SE; Serosa; SM, submucosa; SS, subserosa; T, transverse colon

CLMSs can develop from both the MM and MP membranes, but developed from MM is uncommon.^5)^ There were 10 of pathological (p) MP cases that indicated CLMS arising from the MP membrane (Cases 18,^13)^ 31,^22)^ 34,^24)^ 36,^25)^ 38,^27)^ 40,^29)^ 46,^34)^ 48,^36)^ 49,^37)^ 51^39)^; **Table 1**), and 5 pSM cases (Cases 16,^13)^ 35,^5)^ 37,^26)^ 45,^33)^ and ours; **Table 1**) that arose from MM. **Table 2** summarizes the clinicopathological characteristics of pSM and pMP CLMS cases. The results showed that all pSM cases had not developed recurrent tumors and were alive, with a longer follow-up period. Wang et al. reported that smaller tumor size (<8 cm) and younger age (<60 years) were favorable factors for better survival in CLMS patients.^40)^ According to the summary (**Table 2**), the developing membrane of CLMS is assumed to be associated with its malignant aggressiveness and the survival of CLMS patients. Therefore, the histological diagnosis of which membrane CLMS has arisen from is very important and beneficial for predicting the clinical outcomes of the disease. This prognostic factor has never been reported in previous manuscripts.

**Table 2:** Summary of pSM and pMP mural involvement cases of CLMSs’ clinicopathological characteristics

	pSM (N = 5)	pMP (N = 10)
Tumor size (cm, medium)	3 (1–4.5)	6.25 (3.5–23)
Lymph node metastasis (presence, %)	1 (20)	3 (27)
Distant metastasis (presence, %)	0	1 (9)
Recurrence (presence, %)	0	1 (9)
F/U period (month, medium)	20.5 (6–60)	8 (2–18)
Dead (presence, %)	0	2 (18)

CLMS, colon leiomyosarcoma; F/U, follow up; MP, muscularis propria; N, number; p, pathological; SM, submucosa

Surgery is considered the standard treatment for CLMS, but the significant of LN dissection is still unknown because lymph node metastasis of CLMSs is rather uncommon.^27)^ However, there were 3 cases of localized disease with LN metastasis (Cases 16,^13)^ 38,^27)^ 51^39)^; **Table 1**) and 1 case of heterochronic LN recurrence (Case 39^28)^; **Table 1**). CLMSs frequently behave aggressively,^7)^ so it seems to be better to perform LN dissection for curative surgery, similar to the approach for colorectal adenocarcinoma.

Smrke et al. showed that recurrence is a significant risk factor for poor prognosis even with complete oncological resection.^41)^ In the reviewed 52 cases (**Table 1**), the liver was the most common site of metastasis site (8 cases), and the recurrence sites were of equivalent frequency in the liver and lung (6 and 4 cases, respectively). Most of them received doxorubicin and/or ifosfamide treatment according to standard therapy for LMSs. However, the effectiveness of chemotherapy is unclear for distant metastasis and recurrence cases of CLMS. Some literature reported cases that underwent radical resection for distant lesions and/or recurrence sites.^19,37,38)^ Those reports suggested that long-term survival was achieved after wide surgical excision with negative margins. Radical resection possibly contributes to prolonged survival in patients with metastasis and recurrence diseases.

Several cases received adjuvant chemotherapy after curative surgery (Case 13,^11)^ 19,^14)^ 25,^19)^ 28,^19)^ 43^31)^, 48^36)^; **Table 1**); however, the benefits of adjuvant chemotherapy for CMLS are still controversial because of inadequate follow-up time and limited case numbers.

In the present case, she was older than 60 years, but the tumor size was smaller than 8 cm and it was a pSM tumor. Considering there was no evidence supporting adjuvant chemotherapy, we decided to select careful and short-term observation without adjuvant chemotherapy. She has been alive 24 months after the surgery.

## CONCLUSIONS

This literature demonstrates CLMS arising from MM and suggests that pathological diagnosis is associated with prognosis, as shown by a review of previously reported cases.

## DECLARATIONS

### Funding

This manuscript was not funded externally.

### Authors’ contributions

Y Kasagi and MS wrote the manuscript.

MS, TN, and AI performed the surgery.

Y Kasagi and MM participated in the surgery.

Y Kasagi, MS, TN, RY, MT, NK, AI, Y Kimura, and MM participated in the study design and coordination.

Y Koga and KT developed the histological staining and diagnosed the findings.

MS and Y Kasagi performed the overall organization of writing the manuscript.

All authors read and approved the final manuscript.

### Availability of data and materials

Data sharing is not applicable to this article, as no datasets were generated or analyzed during the current study.

### Ethics approval and consent to participate

This work does not require ethical considerations or approval, and informed consent to participate in this study was obtained from the patient.

### Consent for publication

Consent was obtained from the patient and the patient’s family for the publication of this case report and accompanying images.

### Competing interests

The authors declare that they have no competing interests.

## References

[1] Aggarwal G Sharma S Zheng M Primary leiomyosarcomas of the gastrointestinal tract in the post–gastrointestinal stromal tumor era. Ann Diagn Pathol 2012; 16: 532–40. 22917807 10.1016/j.anndiagpath.2012.07.005

[2] Miettinen M Sarlomo-Rikala M Sobin LH Gastrointestinal stromal tumors and leiomyosarcomas in the colon. A clinicopathologic, immunohistochemical, and molecular genetic study of 44 cases. Am J Surg Pathol 2000; 24: 1339–52. 11023095 10.1097/00000478-200010000-00003

[3] Hirota S Isozaki K Moriyama Y Gain-of-function mutations of c-kit in human gastrointestinal stromal tumors. Science 1998; 279: 577–80. 9438854 10.1126/science.279.5350.577

[4] Kono M Tsuji N Ozaki N Primary leiomyosarcoma of the colon. Clin J Gastroenterol 2015; 8: 217–22. 26208828 10.1007/s12328-015-0584-9

[5] Yahagi M Ishii Y Hara A Laparoscopic surgery to treat leiomyosarcomas of the sigmoid colon: a case report and literature review. Surg Case Rep 2019; 5: 20. 30756192 10.1186/s40792-019-0579-8 PMC6372699

[6] Buxton RW. Smooth muscle tumors of the gastrointestinal tract. Am Surg 1960; 26: 666–77. 13689510

[7] Warkel RL Stewart JB Temple AJ. Leiomyosarcoma of the colon: report of a case and analysis of the relationship of histology to prognosis. Dis Colon Rectum 1975; 18: 501–6. 1181152 10.1007/BF02587219

[8] Insabato L Di Vizio D Ciancia G Malignant gastrointestinal leiomyosarcoma and gastrointestinal stromal tumor with prominent osteoclast-like giant cells. Arch Pathol Lab Med 2004; 128: 440–3. 15043462 10.5858/2004-128-440-MGLAGS

[9] Michalopoulos A Papadopoulos VN Basdanis G Colorectal gastrointestinal mesenchymal tumours. Report of a stromal case of the rectum (GIST) and a leiomyosarcoma of the transverse colon. Tech Coloproctol 2004; 8(Suppl 1): s155–7. 15655606 10.1007/s10151-004-0142-9

[10] Agaimy A Wünsch HP. True smooth muscle neoplasms of the gastrointestinal tract: morphological spectrum and classification in a series of 85 cases from a single institute. Langenbecks Arch Surg 2007; 392: 75–81. 17021790 10.1007/s00423-006-0092-y

[11] Resch T Oberhuber R Zitt M Leiomyosarcoma of the colon: unresolved issues of a rare but highly aggressive malignancy. Am Surg 2011; 77: E62–4. 21679535

[12] Hamai Y Hihara J Emi M Leiomyosarcoma of the sigmoid colon with multiple liver metastases and gastric cancer: a case report. BMC Gastroenterol 2012; 12: 98. 22849696 10.1186/1471-230X-12-98 PMC3507816

[13] Yamamoto H Handa M Tobo T Clinicopathological features of primary leiomyosarcoma of the gastrointestinal tract following recognition of gastrointestinal stromal tumours. Histopathology 2013; 63: 194–207. 23763337 10.1111/his.12159

[14] Yaren A Değirmencioğlu S Callı Demirkan N Primary mesenchymal tumors of the colon: a report of three cases. Turk J Gastroenterol 2014; 25: 314–8. 10.5152/tjg.2014.4010 25141322

[15] Abdel Samie A Sun R Fayyazi A Leiomyosarcoma of the Sigmoid Colon: a rare cause of intestinal intussusception. J Gastrointest Cancer 2014; 45 (Suppl 1): 6–9. 23812956 10.1007/s12029-013-9520-8

[16] Granero-Peiró L Martínez-Ortega P Sánchez-Justicia C Leiomyosarcoma of the ascending colon: a rare tumor with poor prognosis. Rev Esp Enferm Dig 2015; 107: 584–5. 26334473

[17] Kiran P Shiny MP Dhanya SK Diagnosis of leiomyosarcoma of colon. J Cancer Res Ther 2015; 11: 1035. 10.4103/0973-1482.154017 26881636

[18] Janevski V Selmani R Janevska V Leiomyosarcoma of the colon. Med Pregl 2015; 68: 413–7. 26939310 10.2298/mpns1512413j

[19] Faraj W El-Kehdy J Nounou GE Liver resection for metastatic colorectal leiomyosarcoma: a single center experience. J Gastrointest Oncol 2015; 6: E70–6. 26487954 10.3978/j.issn.2078-6891.2015.044 PMC4570914

[20] Kim VM Goicochea L Fang HS. Case report: collision tumour of colon leiomyosarcoma and adenocarcinoma. J Clin Diagn Res 2016; 10: PD03–4. 10.7860/JCDR/2016/16949.7956 PMC4963706 27504346

[21] Akutsu D Mizokami Y Suzuki H A rare case of colonic leiomyosarcoma in association with ulcerative colitis. Intern Med 2016; 55: 2799–803. 27725539 10.2169/internalmedicine.55.6770 PMC5088540

[22] Jideh B Yang T Turner I. Rectal bleeding due to leiomyosarcoma. Clin Gastroenterol Hepatol 2017; 15: e1–2. 27530097 10.1016/j.cgh.2016.08.008

[23] Yang J. Primary leiomyosarcoma in the colon; a case report. Medicine (Baltimore) 2018; 97: e9923. 29443772 10.1097/MD.0000000000009923 PMC5839827

[24] Crystal JS Korderas K Schwartzberg D Primary leiomyosarcoma of the colon: a report of two cases, review of the literature, and association with immunosuppression for IBD and rheumatoid arthritis. Case Rep Surg 2018; 2018: 6824643. 29780656 10.1155/2018/6824643 PMC5892970

[25] Devriendt S Leman G Vanrykel F. Primary leiomyosarcoma of the colon: a case report and review of the literature. Acta Chir Belg 2020; 120: 353–6. 30879400 10.1080/00015458.2019.1589185

[26] Merichal Resina M Cerdan Santacruz C Sierra Grañón E Leiomyosarcoma of the colon. A very uncommon condition with poor prognosis. (Article in English, Spanish). Gastroenterol Hepatol 2020; 43: 200–1. 31864684 10.1016/j.gastrohep.2019.11.002

[27] Tago T Suzuki S Kuroda J Leiomyosarcoma of the transverse colon with lymph node metastasis and malignant transformation: a case report. Surg Case Rep 2020; 6: 256. 33006746 10.1186/s40792-020-00998-4 PMC7532233

[28] Beauchamp A Hajjar R Khullar S Mesenteric lymph node recurrence of a primary colorectal leiomyosarcoma. Case Rep Surg 2020; 2020: 6935834. 32257499 10.1155/2020/6935834 PMC7125469

[29] Wong YC Chan SY Yuen KY Locally invasive and obstructive colonic leiomyosarcoma: a diagnostic and therapeutic challenge. Hong Kong Med J 2020; 26: 73–5. 32077864 10.12809/hkmj197873

[30] Bananzadeh A Mokhtari M Sohooli M Two cases of primary leiomyosarcoma of sigmoid colon treated with laparoscopic surgery: a case report and a review of literature. Int J Surg Case Rep 2021; 87: 106420. 34543950 10.1016/j.ijscr.2021.106420 PMC8455635

[31] Kim Y Jung YY Kim EK. Leiomyosarcoma of the sigmoid colon causing sigmoido-rectal intussusception: a case report. J Korean Soc Radiol 2021; 82: 201–6. 10.3348/jksr.2020.0038 PMC9432394 36237468

[32] Al Laham O Albrijawy R Atia F Spindle cell sarcoma (SCS); a case of primary leiomyosarcoma (LMS) of the sigmoid colon presented as intestinal obstruction. J Surg Case Rep 2021; 2021: rjab515. 34876975 10.1093/jscr/rjab515 PMC8643466

[33] Wong GS Yudina SV Reyes MCD. Laparoscopic right hemicolectomy to curatively treat primary leiomyosarcoma at the ileocecal valve. ACG Case Rep J 2021; 8: e00670. 34646904 10.14309/crj.0000000000000670 PMC8500562

[34] Nguyen C Athigaman M Qureshi A. Giant leiomyosarcoma of the transverse colon. BMJ Case Rep 2021; 14: e246646. 10.1136/bcr-2021-246646 PMC8640626 34857593

[35] Lugo-Fagundo E Fishman EK. Colorectal leiomyosarcoma: a case report. Radiol Case Rep 2022; 17: 2812–4. 35694635 10.1016/j.radcr.2022.05.023 PMC9184291

[36] Pagliai L Annicchiarico A Morini A The double challenge (preoperative diagnosis and surgical approach) of primary leiomyosarcoma of the sigmoid colon. Acta Biomed 2022; 93(S1): e2022124. 35421072 10.23750/abm.v93iS1.11652 PMC10510961

[37] Massaras D Kontis E Stamatis K Primary leiomyosarcoma of the colon with synchronous liver metastasis. Rare Tumors 2022; 14: 20363613221080549. 35360880 10.1177/20363613221080549 PMC8961372

[38] Lee SH Bae SH Lee SC Curative resection of leiomyosarcoma of the descending colon with metachronous liver metastasis: a case report. World J Gastrointest Surg 2023; 15: 992–9. 37342841 10.4240/wjgs.v15.i5.992 PMC10277942

[39] Jenkins P Joiner M Kumar A High grade leiomyosarcoma of the transverse colon with positive lymph node metastasis: to treat or not to treat with adjuvant radiation therapy? BMJ Case Rep 2023; 16: e253466. 10.1136/bcr-2022-253466 PMC10314409 37336627

[40] Wang Y Wang H Yuan ZL A pooled analysis of risk factors of surgically treated leiomyosarcoma of the colon in adults. World J Surg Oncol 2020; 18: 61. 32222151 10.1186/s12957-020-01838-3 PMC7103068

[41] Smrke A Benson C Strauss DC Gastrointestinal leiomyosarcoma demonstrate a predilection for distant recurrence and poor response to systemic treatments. Eur J Surg Oncol 2021; 47: 2595–601. 33966946 10.1016/j.ejso.2021.04.043

